# Fabrication of potato-like silver molybdate microstructures for photocatalytic degradation of chronic toxicity ciprofloxacin and highly selective electrochemical detection of H_2_O_2_

**DOI:** 10.1038/srep34149

**Published:** 2016-09-27

**Authors:** J. Vinoth Kumar, R. Karthik, Shen-Ming Chen, V. Muthuraj, Chelladurai Karuppiah

**Affiliations:** 1 Department of Chemistry, VHNSN College, Virudhunagar–626001, Tamilnadu, India; 2 Department of Chemical Engineering, National Taipei University of Technology, No. 1, Section 3, Chung-Hsiao East Road, Taipei 106, Taiwan, ROC; 3 Department of Chemistry, National Taiwan University, No. 1, Sec. 4, Roosevelt Rd, Daan District, Taipei, Taiwan-10617.

## Abstract

In the present work, potato-like silver molybdate (Ag_2_MoO_4_) microstructures were synthesized through a simple hydrothermal method. The microstructures of Ag_2_MoO_4_ were characterized by various analytical and spectroscopic techniques such as XRD, FTIR, Raman, SEM, EDX and XPS. Interestingly, the as-prepared Ag_2_MoO_4_ showed excellent photocatalytic and electrocatalytic activity for the degradation of ciprofloxacin (CIP) and electrochemical detection of hydrogen peroxide (H_2_O_2_), respectively. The ultraviolet-visible (UV-Vis) spectroscopy results revealed that the potato-like Ag_2_MoO_4_ microstructures could offer a high photocatalytic activity towards the degradation of CIP under UV-light illumination, leads to rapid degradation within 40 min with a degradation rate of above 98%. In addition, the cyclic voltammetry (CV) and amperometry studies were realized that the electrochemical performance of Ag_2_MoO_4_ modified electrode toward H_2_O_2_ detection. Our H_2_O_2_ sensor shows a wide linear range and lower detection limit of 0.04–240 μM and 0.03 μM, respectively. The Ag_2_MoO_4_ modified electrode exhibits a high selectivity towards the detection of H_2_O_2_ in the presence of different biological interferences. These results suggested that the development of potato-like Ag_2_MoO_4_ microstructure could be an efficient photocatalyst as well as electrocatalyst in the potential application of environmental, biomedical and pharmaceutical samples.

Nowadays, antibiotics are main class of antimicrobial drugs in today’s medicine and widely used to prevent the bacterial infection for both human and animals. In particular, ciprofloxacin (CIP), as an antibiotic drug, plays an important role for the treatment of urinary, digestive infections and pulmonary diseases^1^. The CIP can be entered into the aquatic environment through the intentional disposal of surplus drugs, the release of excreta from human and animals, malapropos treatment in the hospitals or in pharmaceutical industries, improper removal of waste water treatment plants and the use of animal’s feces as agricultural fertilizers^2^,^3^. Probably, the CIP drug is not completely metabolized and the continuous release of CIP into the environments displays the chronic toxicity to bacteria, which causes toxicity to the microorganism and retarding to aquatic vertebrates^4^,^5^. It also interacts with photosynthesis process and inhibits the growth of spinach plants^6^ and cause antibiotic-resistance bacteria growth in the environment^7^. Therefore, the removals of CIP from the water sources are major concern to protect the aquatic system and soil environment. Several methods have been reported for the removal of CIP from water including adsorption^8^, oxidation^9^, sonolysis^10^, sorption^11^, (photo)-Fenton process^12^, photocatalytic degradation^13^,^14^,^15^ and ozonation^16^. Among them, photocatalysis is an efficient, cost-effective and eco-friendly method for the environmental remediation, which degrades the hazardous organic pollutants into easily bio-degradable or nontoxic molecules^17^,^18^.

On the other hand, hydrogen peroxide (H_2_O_2_) is an essential intermediate in the various food manufacturing and also involved in our life process. The detection of H_2_O_2_ is a paramount issue because it is a major reactive oxygen species, generated by most oxidases in mitochondria and related to the several bodily disorders such as myocardial infarction, atherosclerosis, and Alzheimer’s disease, cancer, etc.^19^. H_2_O_2_ is also acts contrarily in cell growth, differentiation, physiological signaling pathways, migration and immune function system^20^. Moreover, it is most significant chemical in clinical, pharmaceutical industries and atomic power stations, which dramatically have an effect on the cloud and rainwater^21^ as well as a precursor to the formation of more reactive and potentially harmful hydroxyl radicals^22^. Therefore, the trace level detection of H_2_O_2_ is an important concern for the environment and medicinal fields. Up to now, various methods are available to detect the H_2_O_2_ and some of them have been explored a satisfactory results to the concern of H_2_O_2_ detection^23^. However, the electrochemical methods hold more advantages such as simplicity, portability, rapid analysis, high sensitivity and low-cost instrumentation. Due to these attractive features, the electrochemical methods are represent a promising alternative technique for the determination of H_2_O_2_.

Transition metal-based molybdates (M = Fe, Ni, Co, Ag, Mn etc.,) are considered as an important inorganic material which are widely explored in different applications such as Li-ion storage batteries^24^, birefringent filters^25^, supercapacitors^26^,^27^, optical fibers^28^, photoluminescence^29^, scintillation crystal^30^, photocatalyst^31^,^32^, humidity sensors^33^, magnetic properties^34^ and catalysts^35^. However, low dimensional metal molybdates have attracted more curiosity in current years. In particular, silver molybdate (Ag_2_MoO_4_) has attracted considerable attention because of its unique properties such as environmental friendly, photoluminescence, high electrical conductivity, excellent antimicrobial activity, good photocatalytic activity and remarkable electrochemical energy storage performance^36^. Due to these properties, the Ag_2_MoO_4_ is potentially used in several applications including ion-conducting glasses^37^, high-temperature lubrication^38^, gas sensor^39^, antibacterial material^40^, photoswitches^41^ and ceramics^42^. In photocatalysis, Ag_2_MoO_4_ has paid significant attention owing to it’s photosensitivity which make this material with high photocatalytic activity under UV or visible-light irradiation. Recently, few kinds of literature are reported based on Ag_2_MoO_4_ and its composite that act as a photocatalyst for the degradation of organic dyes into the wastewater^43^,^44^,^45^. The photocatalytic activity mainly depends on the crystal and electronic structures of materials that affect the energy band structure and the efficiency of charge carrier transfer. Moreover, to improve their physicochemical properties of the photocatalyst, researchers have growled a number of approaches to obtain the different morphologies of Ag_2_MoO_4_ including nanoparticles, nanorods, nanowires, wire-like nanostructures, nanoclusters, broom-like, flower-like microstructures and microcrystals^36^,^41^,^46^,^47^,^48^,^49^,^50^. However, to the best of our knowledge, we reported the synthesis of potato-like Ag_2_MoO_4_ microstructure, its applications for the photocatalytic degradation of CIP and electrochemical detection of H_2_O_2_ for the first time.

In this present work, we developed a simple one-pot hydrothermal synthesis of potato-like Ag_2_MoO_4_ microparticles with the assistance of urea and characterized using various analytical and spectroscopic techniques in detailed and further evaluated for electrochemical sensing and photocatalytic applications, as illustrated Fig. 1. Fascinatingly, we find that the as-prepared potato-like Ag_2_MoO_4_ microparticles exhibited a high-performance electrochemical sensor for the detection of H_2_O_2_. Moreover, their photocatalytic activity towards the removal of CIP antibiotic into the environment was also investigated with efficient degradation rate.

## Results and Discussion

**Figure 2:**
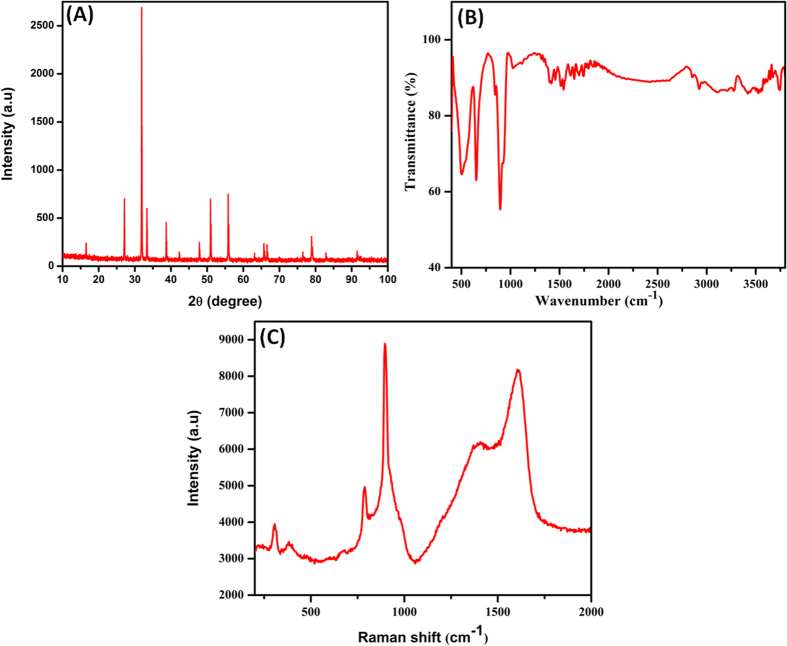
(**A**) XRD patterns (**B**) FTIR spectra and (**C**) Raman spectra of as-prepared Ag_2_MoO_4_.

The crystalline structure and phase purity of the as-synthesized samples were determined by using XRD pattern as shown in Fig. 2A. The distinctive diffraction peaks obtained at 16.48°, 27.09°, 31.87°, 33.31°, 38.65°, 42.27°, 47.84°, 50.93°, 55.81°, 63.10°, 65.70°, 66.57°, 76.49° and 78.90° in the 2θ range which well agreed to the (111), (220), (311), (222), (400), (331), (422), (511), (440), (620), (533), (622), (642) and (731) reflection planes, respectively. Aforementioned planes are well related to the standard XRD report of cubic phase structured Ag_2_MoO_4_ with the space group of Fd
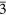
m^51^. From the XRD pattern, it was clearly revealed that the as-synthesized product is β-Ag_2_MoO_4_^36^. The appearance of sharp and high intense peaks demonstrated the higher crystalline nature of cubic Ag_2_MoO_4_ phase. There is no any other peaks were appeared which related to the Ag_2_O or MoO_3_ phase, indicates the as-synthesized Ag_2_MoO_4_ was homogeneous solid.

FTIR and Raman spectroscopy is an important tool for analyzing the involvement of functional groups present in the as-synthesized Ag_2_MoO_4_. In the FTIR spectra (Fig. 2B), the absorption peaks at 3280 and 1650 cm^−1^ correspond to the O-H stretching and bending vibrations of the water molecules, respectively^52^. The peak at 645 cm^−1^ is confirmed the Ag-O stretching vibration of Ag_2_MoO_4._ The sharp peak at 891 cm^−1^ attributed to the anti-symmetric Mo-O stretching in tetrahedral MoO_4_^2−^ ion^53^. Raman spectra showed (Fig. 2C) the peaks at 896, 782, 382 and 305 cm^−1^ which are due to the ν_1_ (A_g_), ν_3_ (E_g_), ν_4_ (B_g_) and ν_2_ (A_g_) symmetric and asymmetric stretching vibration modes of Ag_2_MoO_4_, respectively. The vibration modes at 896 and 782 cm^−1^ could be ascribed to the symmetric stretching vibration of Mo-O bond of the MoO_4_ unit and the asymmetric stretching vibrations of the molybdate ion, respectively. The peaks at 382 and 305 cm^−1^ were clearly indicated the ν_4_ and ν_2_ bending vibration modes of tetrahedral MoO_4_^54^.

**Figure 3:**
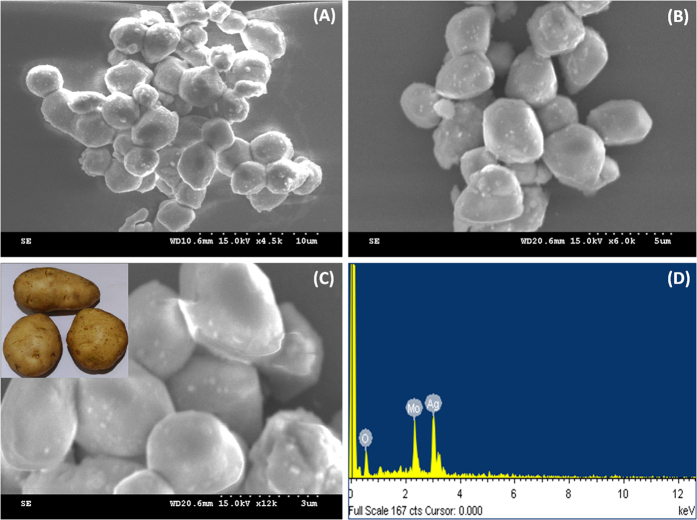
(**A–C**) SEM images of Ag_2_MoO_4_ at different magnifications (**A**) 10 μm (**B**) 5 μm (**C**) 3 μm and (**D**) corresponding to EDX spectrum of Ag_2_MoO_4_.

The surface morphology of the as-prepared microstructures was investigated by SEM. Figure 3(A–C) illustrated the general views of the different magnifications of the obtained Ag_2_MoO_4_ microstructures. The images are clearly displayed the potato-like structure of Ag_2_MoO_4_ which seems like bunches of potatoes with clean and fairly smooth surfaces, the average diameters of microstructures about 1–2 μm. Energy dispersive x-ray spectra (EDX) were used to identify the elements present in the as-prepared Ag_2_MoO_4_ microparticles, as depicted in Fig. 3D. The EDX spectra showed the peaks at approximately 0.5, 2.4 and 3 keV reveal the presence of O, Mo, and Ag elements in the material without any other significant impurities.

**Figure 4:**
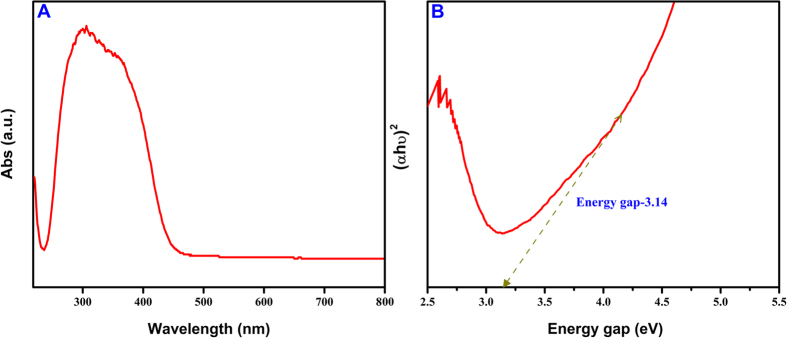
(**A**) UV-Vis diffuse reflectance spectra (DRS) and (**B**) Energy gap spectra of Ag_2_MoO_4_.

The bandgap energy of the Ag_2_MoO_4_ is an important parameter for the selection of suitable kind of light source needed for the degradation purposes. The UV-Vis (Diffuse reflectance) absorption spectrum of Ag_2_MoO_4_ microparticles is shown in Fig. 4A. The results shows that the relation between the normalized absorption of the photocatalyst and wavelength with a range of 200–800 nm. The most part of absorption spectra of Ag_2_MoO_4_ falls in the UV region, a broad steep from 300 to 420 nm which corresponds to the band gap energy value from 3–3.34 eV. The band gap value was calculated using Tauc’s equation and the graph plotted (αhν)^2^ against (hν) as can be seen in Fig. 4B. The calculated band gap energy value is 3.14 eV. On the other hand, the bandgap of Ag_2_MoO_4_ is significantly altered compared to that of previous reports^36^,^43^,^44^,^50^. The oxygen vacancy created in the crystal lattice of the Ag_2_MoO_4_ is leads to the distortion in the energy levels and influenced the bandgap which may be attributed to the effect of hydrothermal environment on the surface microstructures.

**Figure 5:**
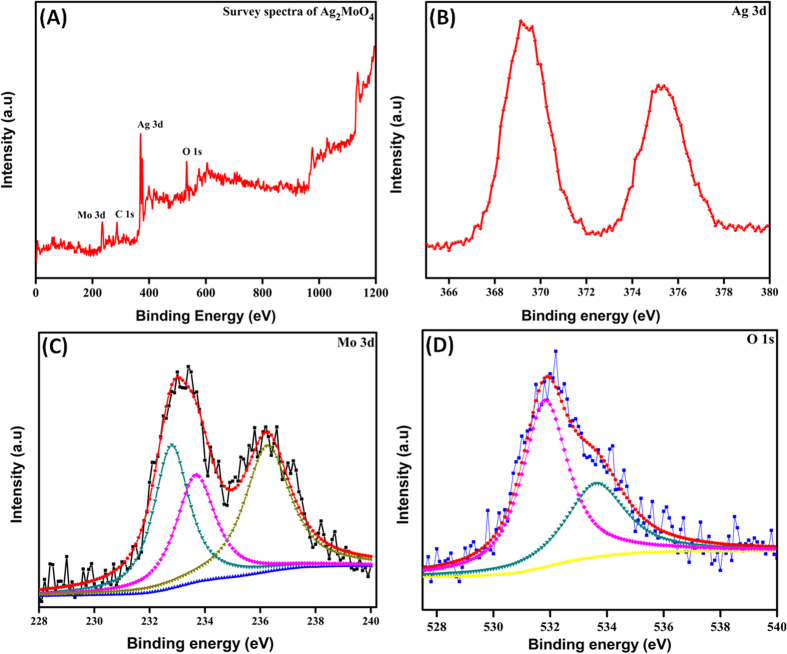
(**A**) XPS survey spectra of Ag_2_MoO_4,_ (**B–D**) High resolution XPS spectra of Ag 3d, Mo 3d and O 1s.

X-ray photoelectron spectroscopy (XPS) was used to evaluate the information about the chemical composition and chemical status of the as-synthesized Ag_2_MoO_4_, as shown in Fig. 5. The overall XPS spectrum in Fig. 5A shows the coexistence of elements Mo, C, Ag and O within the as-prepared Ag_2_MoO_4_ microparticles and no other impurities were detected, which are in good agreement with EDX report. In addition, the presence of C peak at 284.9 eV is ascribed to the adventitious hydrocarbon from the XPS instrument and it is inherent. High resolution scanning XPS spectra clearly confirms the Ag 3d, Mo 3d, and O 1s level, which is fitted by using the Gaussian fitting method, as shown in Fig. 5(B–D). In Fig. 5B, the Ag 3d spectra displays the two peaks at 368.7 and 374.4 eV attributed to the Ag 3d_5/2_ and Ag 3d_3/2_ electron binding energy in Ag_2_MoO_4_, respectively^55^. The peaks at 233.2 and 236.3 eV ascribed to the Mo 3d_5/2_ and Mo 3d_3/2_ binding energy of Mo 3d, respectively. The major binding energy peaks Mo 3d_5/2_ and Mo 3d_3/2_ are separated by 3.1 eV, which belongs to the Mo^6+^ oxidation state as depicted in Fig. 5C^56^. The high intense peaks at around in the range of 530.5–533.6 eV revealed the presence of O 1s core level^57^ in Ag_2_MoO_4_ (Fig. 5D). Hence, the obtained XPS results clearly confirmed that the valence of Ag, Mo and O are +1, +6 and −2, respectively, which is very good agreement with the phase structure of Ag_2_MoO_4_.

**Figure 6:**
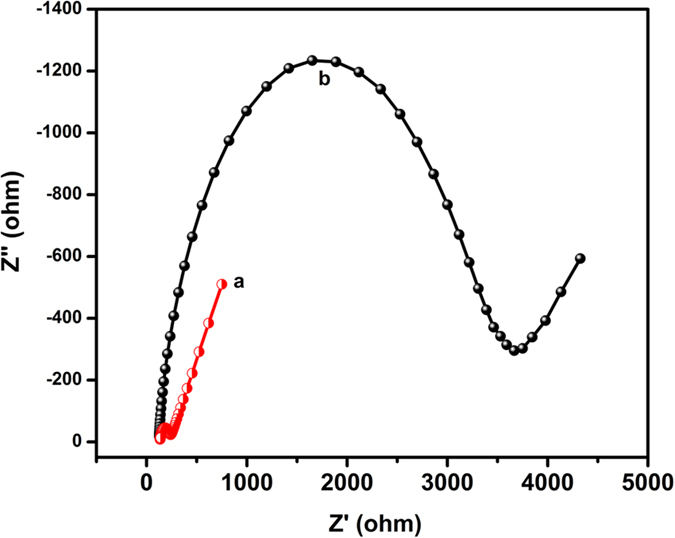
Electrochemical impedance spectroscopy of different modified electrodes (**a**) bare GCE (**b**) Ag_2_MoO_4_ modified GCE in 0.1 M KCl solution containing 5 mM [Fe(CN)_6_]^3−/4−^.

Electrochemical impedance spectroscopy (EIS) was used to investigate the changes of the electrode surface during the fabrication process. The nyquist curves of the EIS spectra was observed using bare GCE (a) and Ag_2_MoO_4_ modified GCE (b) in 0.1 M KCl containing 5.0 mM K_3_Fe(CN)_6_/K_4_Fe(CN)_6_ (Fig. 6). The diameter of the semicircle indicates the electron transfer resistance (R_ct_) of the electrode. This resistance controls the electron transfer kinetics of redox probe at the electrode interface. From the EIS results, the Ag_2_MoO_4_ modified GCE (b) shows larger semicircle than bare GCE (a) reveals that the Ag_2_MoO_4_ modified GCE can increase the electron transfer resistance on electrode surface, because it is hindered the electron transfer of K_3_Fe(CN)_6_/K_4_Fe(CN)_6_, thus confirming the successful modification of Ag_2_MoO_4_ on the GCE surface.

### Photocatalytic activity

**Figure 7:**
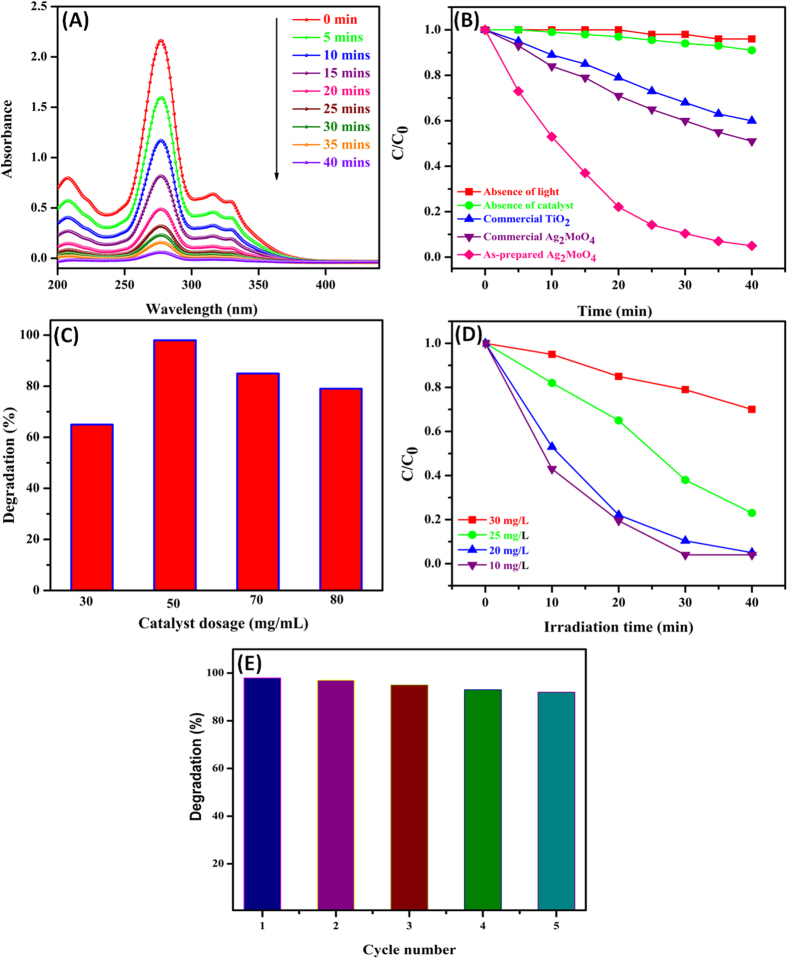
(**A**) Absorption spectrum of CIP in the presence of 50 mg/mL Ag_2_MoO_4_ under UV-light illumination. (**B**) Photodegradation of CIP in the presence of different catalysts. (**C**) Effect of catalyst amount dosage on the photodegradation of CIP. (**D**) Effect of initial CIP concentration on the photodegradation and (**E**) reusability of the potato-like Ag_2_MoO_4_ photocatalyst.

The photocatalytic behavior of as-prepared Ag_2_MoO_4_ microparticles was performed against the degradation of CIP under UV-light illumination, as illustrated in Fig. 7A. The absorbance spectrum shows the progressive degradation of CIP and the main absorption peak of CIP was observed at 276 nm and other small peaks were also completely diminished within 40 min. The degradation percentage of CIP solution was estimated from the relative intensity of absorbance in UV-visible spectra. The relative intensity of absorbance was decreased and reached almost zero within 40 min, reveals that the Ag_2_MoO_4_ microparticles degraded the 98% of the CIP solution. Initially, the utmost decrement of CIP was observed which could be attributed to the competence of CIP with hydroxyl radicals generated by UV-light photoexcitation of Ag_2_MoO_4_ microparticles. However, as the reaction proceeds, the formation of by-products from degradation might compete with the hydroxyl radicals and adsorption sites on the catalyst surface. Hence, the gradual degradation of CIP was observed.

Under similar degradation conditions, commercially available Ag_2_MoO_4_ and commercial TiO_2_ powder were also employed as a photocatalyst and their accurate comparison was carried out which depicted in Fig. 7B. The photocatalytic activity of commercial Ag_2_MoO_4_ and TiO_2_ on CIP degradation exhibited a poor efficiency and the corresponding degradation percentages are observed around 38% and 32%, respectively. Moreover, there is no significant degradation was observed either in the absence of light or in the absence of a catalyst. The results clearly confirmed the as-synthesized Ag_2_MoO_4_ shown a superior photocatalytic efficacy over the commercial Ag_2_MoO_4_ and TiO_2_ powder for the degradation of CIP solution.

Catalyst dosage is an important parameter that can significantly influence the rate of photodegradation. Figure 7C shows the efficiency of CIP degradation (%) against the effect of catalyst loading by varying the catalyst amount from 30 to 80 mg/mL, wherein the concentration of CIP and intensity of the light were maintained as constant. It can be seen that the rate of photocatalytic degradation was maximum at 50 mg/mL, due to the generation of more number of electron-hole pairs. However, the addition of excess amount of photocatalyst can blocks the existing active sites and interfere with the diffusion of photons. As a result, the photocatalytic degradation of CIP was decreased when increase the concentration of photocatalyst over 50 mg/mL^58^.

Effect of initial concentration of CIP solution on the photodegradation was also investigated and the concentration varied from 10 to 30 mg/L under identical conditions, the results are shown in Fig. 7D. In the present case ~98% degradation was achieved at 20 mg/L whereas 83% and 77% degradation were observed at 25 and 30 mg/L concentration of CIP, respectively. This is due to the screening of light by the CIP solution and the less number of photons to reach the Ag_2_MoO_4_ surface. Hence, the electron-hole pair generation is reduced greatly and consequently, the degradation efficiency decreased.

Generally, the reactive oxidative species (ROS) *viz* hydroxyl radical (·OH), superoxide radical anion (O_2_^·−^), hole (h^+^) and electron (e^−^) involved in the photocatalytic reaction^59^. The photocatalytic mechanism of the degradation of CIP is represented in the following equations


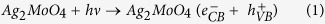



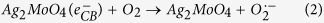



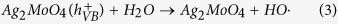



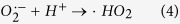



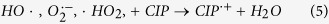



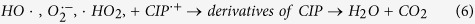


The semiconductor photocatalyst generally undergoes excitation under light illumination with energy greater than the bandgap while the e^−^ excited from the valence band (VB) to the conduction band (CB) leaves h^+^ in the VB^60^. In the present study, Ag_2_MoO_4_ was irradiated by UV light which produced the e^−^ in the CB and h^+^ in the VB, as illustrated in the Eq. 1. The e^−^ in the CB were reacted with the oxygen molecule to form O_2_^·−^ and the O_2_^·−^ was reacted with the water molecule to form ·HO_2_, as shown in Eqs 2 and 4. Furthermore, h^+^ in the VB adsorbed water molecules and reacted to form ·OH, as given in Eq. 2. The ROS formed in the photocatalytic reaction facilitated the degradation of the CIP by the stepwise photocatalytic reduction process (Eqs 5 and 6).

The high photocatalytic activity as well as recycling ability of the catalyst is a vital issue for long-term use in practical applications. To evaluate the sustainability of Ag_2_MoO_4_, the recycling experiments were carried out for the degradation of CIP, as illustrated in Fig. 7E. The photocatalytic activity of the Ag_2_MoO_4_ on CIP degradation did not show a significant loss and attains more than 90% of degradation rate even after the five successive cycles with every cycle lasting for 40 min, which indicating that the potato-like Ag_2_MoO_4_ microstructure is a very effective and highly stable photocatalyst. Thus, the obtained results also indicated a good reusability of the catalyst. Moreover, the poor loss of catalytic activity during the recycle performance is due to the accumulation of impurities on the surfaces of the photocatalyst.

### Electrochemical performance to hydrogen peroxide on Ag_2_MoO_4_ modified GCE

**Figure 8:**
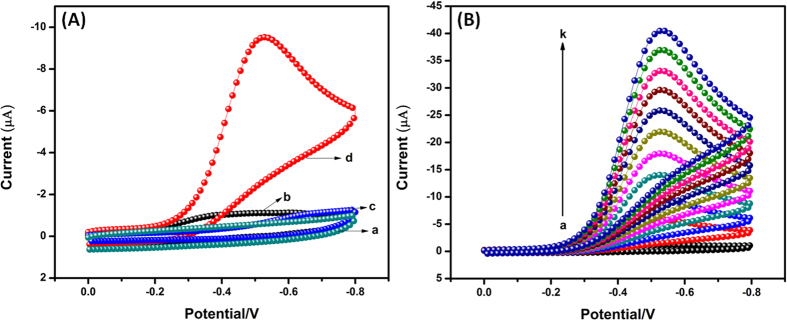
(**A**) Cyclic voltammograms for the reduction of H_2_O_2_ on bare GCE (a,c) and Ag_2_MoO_4_ modified GCE (b,d) in absence (a,b) and presence (c,d) of 200 μM H_2_O_2_ containing N_2_ saturated 0.05 M PBS (pH 7) at a scan rate of 50 mV/s. (**B**) Cyclic voltammograms of Ag_2_MoO_4_ modified GCE in N_2_ saturated 0.05 M PBS (pH 7) in the absence and presence of H_2_O_2_ with different concentrations (a–k: 0 to 1 mM) at a scan rate of 50 mV/s.

Cyclic voltammetry studies of the H_2_O_2_ sensor was demonstrated using bare GCE (a,c) and (b,d) Ag_2_MoO_4_ modified GCE in absence (curve a & b) and presence of 200 μM H_2_O_2_ (curve c & d) containing N_2_ saturated 0.05 M PBS (pH 7) at a scan rate 50 mV/s. In the absence of H_2_O_2_ (curve a & b), the results clearly indicates that there is no significant reduction peak appeared in the selected potential window. Whereas, in the presence of 200 μM H_2_O_2_ on Ag_2_MoO_4_ modified GCE (curve d) shows a strong and higher reduction peak current appeared at the lower onset potential −0.26 V due to the catalytic behavior of Ag_2_MoO_4_. Although, we also observed a slight and not considerable reduction peak in the bare GCE at the longer potential −0.7 V (curve c). The reduction peak current of H_2_O_2_ on Ag_2_MoO_4_ modified GCE is 10 times much higher than that of bare GCE. The above results confirmed that Ag_2_MoO_4_ modified GCE has high catalytic ability for H_2_O_2_ detection. Consequently, Ag_2_MoO_4_ are suitable as mediators to transfer electron between H_2_O_2_ and working electrode and make possible electrochemical regeneration following electron exchange with H_2_O_2_. The possible mechanisms for the electrochemical reduction of hydrogen peroxide as shown in Eqs (7, 8, 9). In order to investigate the electrocatalytic activity of Ag_2_MoO_4_ modified GCE, CVs were performed in the presence of different addition of H_2_O_2_ in 0.05 M PBS (pH 7), as shown in Fig. 8B. The reduction peak current of H_2_O_2_ was linearly increased with increasing the H_2_O_2_ concentration from 0 to 1 mM, which revealing electrocatalytic activity of Ag_2_MoO_4_ modified GCE toward the H_2_O_2_. Furthermore, the low level detection, sensitivity and linear range of H_2_O_2_ were clearly discussed in amperometric (*i-t*) section.


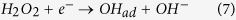



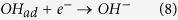



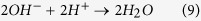


**Figure 9:**
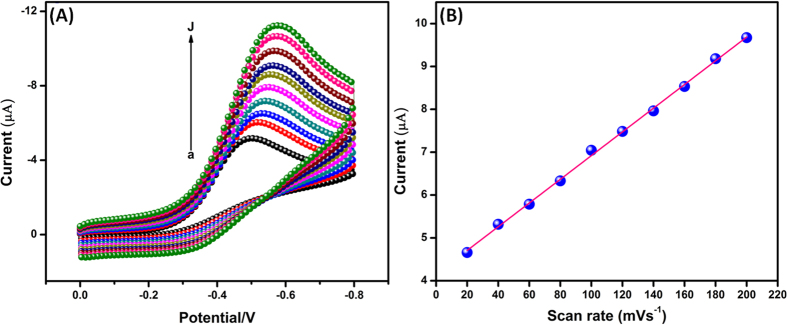
(**A**) Cyclic voltammograms of Ag_2_MoO_4_ modified electrode in 200 μM H_2_O_2_ containing N_2_ saturated 0.05 M PBS (pH 7) at different scan rates from 20 to 200 mV/s (a–j) and (**B**) Corresponding calibration plot of scan rate vs*. I*_pc_ of H_2_O_2_.

Furthermore, the electrocatalytic behavior of the Ag_2_MoO_4_ modified GCE towards H_2_O_2_ was studied with the change of scan rate. Figure 9A reveals the CVs responses of 200 μM of H_2_O_2_ detection at Ag_2_MoO_4_/GCE with different scan rate ranges from 20 to 200 mV/s and its denoted (a–j). When increasing the scan rate from 20 to 200 mV/s, the reduction peak current of H_2_O_2_ was increased and the peak potential was shifted towards the more negative side. The peak current of H_2_O_2_ reduction is directly proportional to the scan rate (Fig. 9B) (Correlation co-efficient R^2^ = 0.998), indicating that the electrode process is surface controlled process^61^.

The effect of pH is very important phenomenon for the electrochemical sensor and biosensor. The electrocatalytic performance of Ag_2_MoO_4_ modified GCE for H_2_O_2_ reduction was examined in different pH solution. The CVs of Ag_2_MoO_4_ modified GCE in the presence of 200 μM H_2_O_2_ at different pH ranges (3 to 11) of 0.05 M PBS solution were studied and its calibration plot is depicted in Figure S1. When the pH value increased from 3 to 5, the reduction peak current increased and then decreased gradually while increasing the pH more than 7. However, higher peak currents are observed at pH 7. Therefore, Ag_2_MoO_4_ modified GCE has good electrocatalytic activity at pH 7 and the reduction of H_2_O_2_ is pH dependence. Therefore, we chosen pH 7 is optimized pH and this pH was used further electrochemical measurements.

### Amperometric determination of H_2_O_2_ at Ag_2_MoO_4_ modified GCE

**Figure 10:**
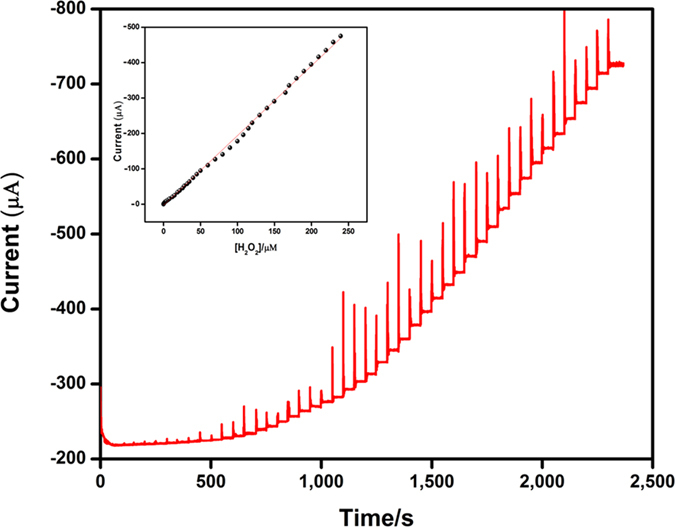
Amperometric *i–t* responses of H_2_O_2_ reduction at Ag_2_MoO_4_ film modified RDGCE upon successive additions of H_2_O_2_ from 0.04 to 247 μM into continuously stirred N_2_ saturated 0.05 M PBS (pH 7). Applied potential: −0.5 V; Rotation rate: 1200 rpm. Inset shows the calibration plot of response current vs. H_2_O_2_ concentration [0.04–240 μM].

The amperometric *i-t* technique is one of the most important method to determine the electrocatalytic activity of modified electrodes in electrochemical sensor and biosensor applications. In the present work, we have utilized an amperometric method to estimate the performance of Ag_2_MoO_4_ modified GCE toward H_2_O_2_ detection. In this regards, Ag_2_MoO_4_ modified rotating disc glassy carbon electrode (RDGCE) was performed in continuously stirred pH 7 solution by applying constant potential at −0.5 V with rotation speed at 1200 rpm. Figure 10 reveals the amperometric *i-t* performance obtained at Ag_2_MoO_4_ modified RDGCE upon the different addition of H_2_O_2_ (0.049 to 247 μM) in the PBS solution. These results undoubtedly shows that Ag_2_MoO_4_ modified film demonstrates a fast and well-defined response obtained in each different addition of H_2_O_2_ concentration. The response time of H_2_O_2_ detection on Ag_2_MoO_4_ modified RDGCE was observed for 5 s, it’s clearly suggesting that the fast electron movement process was occurred in the electrolyte and electrode interface when introducing the H_2_O_2_. The linear response current increases with increasing the concentration of H_2_O_2_ (low concentration to high concentration) from 0.049 to 240 μM (linear range inset; Fig. 10), the obtained sensitivity and limit of detection (LOD) of the sensor is around 9.8 μAμM^−1^ cm^−2^ and 0.03 μM, respectively. The above results suggesting that the Ag_2_MoO_4_ modified RDGC electrode has good electrocatalytic activity towards H_2_O_2_. The analytical parameters, such as LOD, linear range, and sensitivity, of H_2_O_2_ sensor was compared with various modified electrodes are summarized in Table 1. Clearly, the Ag_2_MoO_4_ modified RDGCE, reported here, exhibits good sensitivity and LOD over a wide linear range of H_2_O_2_ concentration compared to other reports^62^,^63^,^64^,^65^,^66^,^67^,^68^,^69^,^70^,^71^,^72^,^73^,^74^,^75^.

**Figure 11:**
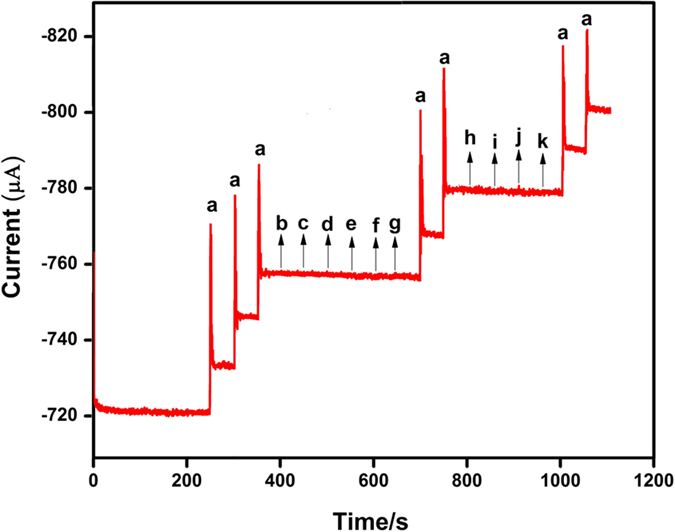
Amperometric *i–t* responses of H_2_O_2_ reduction at Ag_2_MoO_4_ modified RDGCE for the successive addition of 100 μM of H_2_O_2_ (**a**), and 50-fold excess concentration of catechol (**b**), fructose (**c**), lactose (**d**), sucrose (**e**), glucose (**f**), hydroquinone (**g**), ascorbic acid (**h**), uric acid (**i**), dopamine (**j**) and epinephrine (**k**) in continuously stirred N_2_ saturated 0.05 M PBS (pH 7). Applied potential: −0.5 V; Rotation rate: 1200 rpm.

Selectivity is very important study in the electrochemical sensor. In order to investigate selectivity, the proposed sensor was used to detect the H_2_O_2_ in the presence of variety of biological interferences such as catechol (b), fructose (c), lactose (d), sucrose (e), glucose (f), hydroquinone (g), ascorbic acid (h), uric acid (i), dopamine (j) and epinephrine (k) with 50-fold excess concentration of each analytes, as shown in Fig. 11. The Ag_2_MoO_4_ modified RDGCE exhibited well-defined response towards each 100 μM H_2_O_2_ (a). No remarkable responses were monitored for the 50-fold excess concentration of biological interferences as mentioned above. Hence, the Ag_2_MoO_4_ modified RDGCE has excellent selectivity towards the determination of H_2_O_2._ Furthermore, we have studied the stability of our proposed sensor by amperometric (*i-t*) techniques and the results obtained as it can be seen in Figure S2. The prepared sensor retains its 98.2% of its initial response of H_2_O_2_ after prolongs runs up to 2600 s, which suggesting the good stability of the sensor.

In summary, a potato-like Ag_2_MoO_4_ microparticles were successfully prepared through a simple hydrothermal method. Different analytical and spectroscopic methods were used to confirm the structural nature of Ag_2_MoO_4_ microparticles. All the obtained results are strongly evidenced that the prepared compound shows like pristine Ag_2_MoO_4_ without any other impurities. The as-prepared Ag_2_MoO_4_ microparticles explored excellent photocatalytic activity towards the degradation of chronic toxicity CIP under UV-light illumination and 98% of the CIP was degraded within 40 min. On the other hand, the Ag_2_MoO_4_ microparticles were used to fabricate the sensor electrode for the detection of H_2_O_2_ in the potentially interfering biological substances. The Ag_2_MoO_4_ modified GCE revealed a high electrocatalytic activity with wide linear range, good stability and low detection limit than the previous reports. Finally, the Ag_2_MoO_4_ represents an interesting and promising candidate for photocatalytic and electrochemical applications with the advantages of one-pot preparation, unique features, excellent catalytic activity and high sensing performance.

## Experimental Section

### Materials

Silver nitrate (AgNO_3_), sodium molybdate dihydrate (Na_2_MoO_4_.2H_2_O), urea (CH_4_N_2_O), hydrogen peroxide (H_2_O_2_) and all chemicals were purchased from Sigma-Aldrich and used further purification. Ciprofloxacin drug was purchased from Thiruvengadam Medicals, Virudhunagar, India. All other chemicals were of analytical grade and used without further purification. The phosphate buffer solution (PBS) was prepared using 0.05 M Na_2_HPO_4_ and NaH_2_PO_4_ and all the required solutions were prepared using deionized water (DI).

### Synthesis of silver molybdate

In a typical synthesis, 0.5 M of Na_2_MoO_4_.2H_2_O and 0.1 M of AgNO_3_ were dissolved in 60 mL DI water. Then, 0.3 g of urea (10 mL) was gradually added into the above mixture under vigorous stirring at room temperature for 30 min. The mixture was transferred into 100 mL Teflon-lined autoclave and maintained at 120 °C for 8 h in an oven. The autoclave was then cooled down to room temperature naturally, and the obtained yellow product was collected by centrifugation and washed thoroughly with DI water and ethanol for three times and dried overnight at 80 °C.

### Characterization

The powder X-ray diffraction (XRD) analysis was carried out using PANalytical X’Pert PRO X-ray diffractometer measured with Cu-Kα radiation (λ = 1.54178 Å) in the 2θ range of 10–100°. X-ray photoelectron spectroscopy (XPS) measurements were carried out using ULVAC-PHI 5000 VersaProb instrument. Scanning electron microscope (SEM) and Energy dispersive X-ray spectra (EDX) were probed using Hitachi S-3000 H and HORIBA EMAX X-ACT, respectively. Raman spectra were collected in an NT-MDT confocal Raman microscopic system with an exciting laser wavelength of 488 nm and the laser spot-size is around 0.5 μm. Fourier transform infrared spectroscopy (FTIR) was recorded by Shimadzu FT-IR 3000 spectrometer in the diffuse reflectance mode at a resolution of 4 cm^−1^, the sample was pressed into KBr disc with a weight ratio of sample to KBr of 1:100 in the range of 4000-400 cm^−1^. UV-Visible diffused reflectance (DRS) spectrum of the sample was taken from Shimadzu UV-2600 spectrophotometer and BaSO_4_ was used as a reflectance reference material. The absorption spectra in the photocatalytic degradation process were monitored by Shimadzu 2100 UV-Visible spectrometer. The electrochemical impedance spectroscopy (EIS) was performed by XPOT (ZAHNER elektrik instrument). Cyclic voltammetry (CV) and Amperometric (*i-t*) experiments were carried out using CHI 405a work station and PINE instrument, respectively. All the electrochemical studies were carried out in three-electrode cell system, glassy carbon electrode (GCE surface area = 0.071 cm^2^) was used as a working electrode, platinum wire and standard Ag/AgCl electrodes were used as a counter and reference electrode, respectively.

### Photocatalytic activity

The photocatalytic activity of the as-prepared Ag_2_MoO_4_ was evaluated via degradation of CIP solution under UV light illumination (λ = 200~400 nm). In a typical activity, 50 mg/mL of catalyst was dispersed in 100 mL aqueous solutions of CIP (20 mg/L). Prior to illumination, the solution mixture was stirred magnetically for 30 min in the dark to establish the adsorption-desorption equilibrium between CIP and as-prepared Ag_2_MoO_4_ photocatalyst. Then, the solutions were illuminated by UV light (λ_max_ = 365 nm) to induce photocatalytic reaction. During the irradiation, 5 mL of the solution was withdrawn at 5 min time intervals and centrifuged to remove the catalyst. The obtained clear liquor was analyzed by UV-Vis spectrometer to determine the concentration changes of CIP.

### Fabrication of silver molybdate modified GCE

Before surface modification, the GCE was mirror like polished with 0.05 μm alumina slurry and rinsed with DI to remove the alumina particles on the GCE surface. After that the GCE was sonicated for 1 min containing ethanol and water (1:1 ratio). About 5 mg/mL of the as-prepared Ag_2_MoO_4_ was re-dispersed in DI water and about 8 μL (optimized concentration) of the dispersed Ag_2_MoO_4_ was drop casted on the GCE surface. Then it was allowed to dry at room temperature, followed by the dried Ag_2_MoO_4_ modified GCE was rinsed with DI water to remove the loosely attached catalyst on the GCE surface. The obtained Ag_2_MoO_4_ modified GCE was used to further electrochemical experiments. Then, it was stored in room temperature when not in use.

## Additional Information

**How to cite this article**: Kumar, J. V. *et al*. Fabrication of potato-like silver molybdate microstructures for photocatalytic degradation of chronic toxicity ciprofloxacin and highly selective electrochemical detection of H_2_O_2_. *Sci. Rep*. **6**, 34149; doi: 10.1038/srep34149 (2016).

## Supplementary Material

Supplementary Information

## Figures and Tables

**Figure 1:**
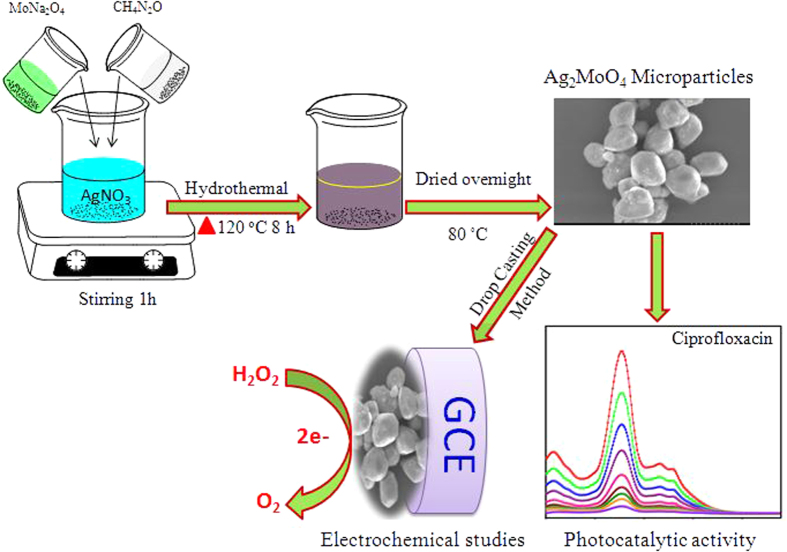
The synthesis route for Ag_2_MoO_4_ microparticles and its application for photocatalytic activity and electrochemical biosensor.

**Table 1:** 

Modified electrode	Method of detection	LR (μM)	LOD (μM)	Ref.
AgNPs/DNA/GCE	Amperometry	2.0–2500	0.6	62
GNs/Ag/GCE	Amperometry	100.0–40,000	28.0	63
AgNWs/CS/GCE	Amperometry	8.0–1350	2.0	64
AgNWs/PtE	Amperometry	0.5–3000	0.2	65
ZnONRs/AgNPs/GCE	Amperometry	0.9–983.0	0.9	66
SiNWs/AgNPs/GCE	Amperometry	0.2 −20,000	0.2	67
AgNPs/GCE	Amperometry	4.0–60.0	1.3	68
PVP–AgNWs/GCE	Amperometry	20.0–3620	2.3	69
GR/AgNWs/GCE	Amperometry	1	1.0	70
CNT/AgNPs/GCE	Amperometry	9.0–9000	1.6	71
RGO/AgNPs/GCE	Amperometry	100.0–8000	7.1	72
Ag/GCE	Amperometry	5.0–12,000	0.5	73
Ag-AlOOH-rGO	Amperometry	5.0 to 4200	1.8	74
Ag-Bt/GCE	Amperometry	—	9.1	75
Ag_2_MoO_4_/GCE	Amperometry	0.04–240	0.03	This work

Comparison of analytical performance of Ag_2_MoO_4_ modified electrode with previously reported similar modified electrodes for the detection of H_2_O_2_. Abbreviations: LR–linear response range; LOD–limit of detection; NPs–nanoparticles; NWs–nanowires; Ag- Silver nanoparticles; CS–chitosan; GCE–glassy carbon electrode; GR–graphene; PVP–polyvinylpyrolidone; GNs–graphene nanosheets; PtE–platinum electrode; CNT–carbon nanotubes; NRs–nanorods; RGO–reduced graphene oxide; AlOOH- Aluminum oxyhydroxide: Ag-Bt- silver nanoparticle-incorporated bentonite clay.
