# Supplementary material for: Fabrication of potato-like silver molybdate microstructures for photocatalytic degradation of chronic toxicity ciprofloxacin and highly selective electrochemical detection of H2O2
Source: Sci Rep. 2016 Sep 27;6:34149. doi: 10.1038/srep34149 (PMC5037444; doi:10.1038/srep34149)
Supplement: Supplementary Information [file srep34149-s1.pdf]

Fabrication of potato-like silver molybdate microstructure for photocatalytic degradation of chronic toxicity ciprofloxacin and highly selective electrochemical detection of H_2O_2

J.Vinoth Kumar[1], R.Karthik[2], Shen-Ming Chen[2*], V. Muthuraj[1] & Chelladurai Karuppiah[2,3]**

[1]Department of Chemistry, VHNSN College, Virudhunagar – 626001, Tamilnadu, India.

[2]Department of Chemical Engineering, National Taipei University of Technology, No. 1, Section 3, Chung-Hsiao East Road, Taipei 106, Taiwan, ROC.

[3]Department of Chemistry, National Taiwan University, No. 1, Sec. 4, Roosevelt Rd, Daan District, Taipei, Taiwan-10617.

*** Authors for Correspondence**

E-mail: smchen78@ms15.hinet.net, Tel: +886 2270 17147, Fax: +886 2270 25238.

E-mail: muthuraj75@gmail.com, Tel: +919940965228

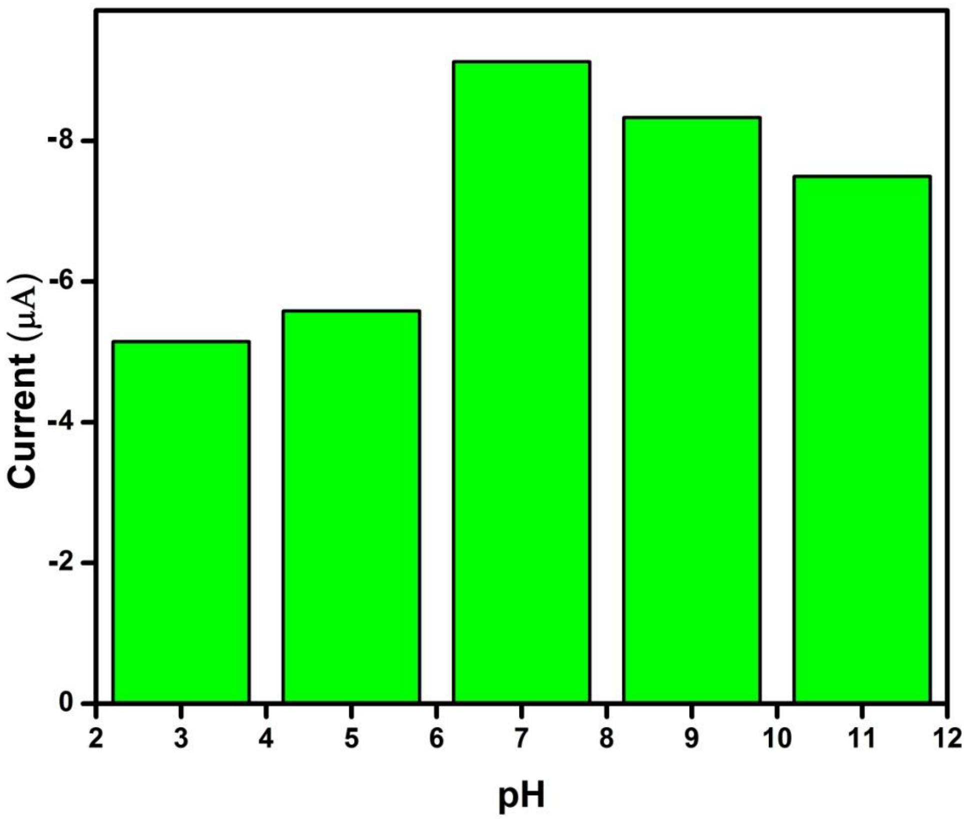

Fig. ***S1*** The calibration plot for effect of pH *vs.* reduction peak current response of 200 µM H_2O_2.

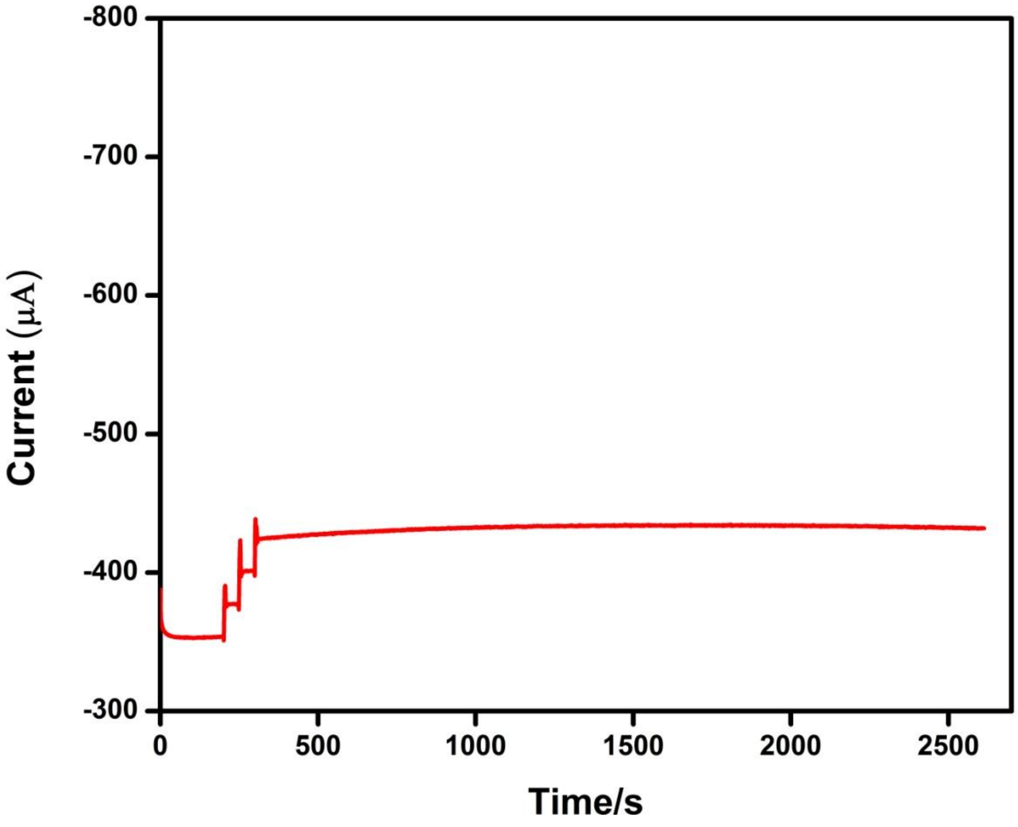

Fig.*S2*. Amperometric i–t response of Ag_2MoO_4 modified RDGCE for addition of 150 μM of H_2O_2 (a) into the constantly stirred N_2 saturated PBS and the background current response up to 2600 s; working potential = -0.5 V.
